# Neutrophil elastase and matrix metalloproteinase 12 in cystic fibrosis lung disease

**DOI:** 10.1186/s40348-016-0053-7

**Published:** 2016-07-25

**Authors:** Claudius J. Wagner, Carsten Schultz, Marcus A. Mall

**Affiliations:** 1 Department of Translational Pulmonology, University of Heidelberg, Heidelberg, Germany; 2 Translational Lung Research Center Heidelberg (TLRC), German Center for Lung Research (DZL), Heidelberg, Germany; 3 Molecular Medicine Partnership Unit (MMPU), University of Heidelberg and European Molecular Biology Laboratory, Heidelberg, Germany; 4 Cell Biology and Biophysics Unit, European Molecular Biology Laboratory, Heidelberg, Germany; 5 Division of Pediatric Pulmonology and Allergy and Cystic Fibrosis Center, Department of Pediatrics, University of Heidelberg, Im Neuenheimer Feld 430, 69120, Heidelberg, Germany

**Keywords:** Airway inflammation, Neutrophil elastase, Matrix metalloproteinase 12, Cystic fibrosis, FRET reporter

## Abstract

Chronic lung disease remains the major cause of morbidity and mortality in patients with cystic fibrosis (CF). Recent studies in young children with CF diagnosed by newborn screening identified neutrophil elastase (NE), a major product released from neutrophils in inflamed airways, as a key risk factor for the onset and early progression of CF lung disease. However, the understanding of how NE and potentially other proteases contribute to the complex in vivo pathogenesis of CF lung disease remains limited. In this review, we summarize recent progress in this area based on studies in βENaC-overexpressing (βENaC-Tg) mice featuring CF-like lung disease and novel protease-specific Förster resonance energy transfer (FRET) sensors for localization and quantification of protease activity in the lung. These studies demonstrated that NE is implicated in several key features of CF lung disease such as neutrophilic airway inflammation, mucus hypersecretion, and structural lung damage in vivo. Furthermore, these studies identified macrophage elastase (matrix metalloproteinase 12 (MMP12)) as an additional protease contributing to early lung damage in βENaC-Tg mice. Collectively, these results suggest that NE and MMP12 released from activated neutrophils and macrophages in mucus-obstructed airways play important pathogenetic roles and may serve as potential therapeutic targets to prevent and/or delay irreversible structural lung damage in patients with CF.

## Introduction

Cystic fibrosis (CF) is a complex disorder affecting multiple epithelial organs that is caused by over 2000 mutations in the cystic fibrosis transmembrane conductance regulator (*CFTR*) gene and remains the most common fatal genetic disease in Caucasian populations [13, 32]. Despite substantial improvements in clinical management, chronic lung disease remains the major cause of morbidity and mortality in patients with CF. Starting as a muco-obstructive lung disease with potentially reversible abnormalities such as airway mucus plugging, intermittent bacterial infection, and inflammation in the first months of life, CF lung diseases invariably progress towards a mucopurulent disorder characterized by chronic infection with specific pathogens such as *Pseudomonas aeruginosa*, non-resolving neutrophilic inflammation and irreversible structural lung damage, ultimately leading to respiratory failure [17, 32]. At the molecular and cellular levels, CF lung disease is caused by abnormal ion transport that disturbs the homeostasis of the thin liquid layer on airway surfaces producing a milieu that renders the airways susceptible for chronic infection and inflammation. CFTR functions as a cAMP-dependent anion (Cl^−^ and bicarbonate) channel and regulator of the amiloride-sensitive epithelial Na^+^ channel ENaC and therefore plays a central role in the regulation of ion/fluid transport across airway epithelia [1, 9, 31, 51]. In CF, CFTR malfunction in the surface epithelium and submucosal glands renders airway surfaces dehydrated and slightly acidic [5, 33, 44]. These defects result in impaired mucus clearance and reduced bacterial killing by antimicrobial peptides thus setting the stage for airway mucus plugging and impaired host defenses that result in a vicious circle of chronic neutrophilic inflammation, infection, and progressive bronchiectasis in CF airways [36].

Because airway neutrophilia is associated with high levels of “free” NE activity leading to a protease-antiprotease imbalance in CF airways, NE has been implicated for a long time as a major player in the pathogenesis of structural lung damage in CF [4, 7, 40, 41, 46, 53]. This concept has been further substantiated by recent studies in infants and young children with CF who were diagnosed by newborn screening and followed longitudinally by annual chest computed tomography (CT) and bronchoalveolar lavage (BAL) [49, 50]. These studies demonstrated that CF lung disease starts in the first months of life, often in the absence of respiratory symptoms, and found a strong association between elevated NE activity in BAL fluid and the onset and progression of structural abnormalities including early bronchiectasis detected by chest CT [49, 50].

While these clinical association studies identified NE as a key risk factor, they do not provide mechanistic insights into how increased NE activity determines the progression of CF lung disease. In this context, it is noteworthy that a series of experimental studies demonstrated that NE has multiple functions that may be either disease promoting or protective in CF airways. Specifically, NE has been implicated in several key features of CF lung disease including airway inflammation, goblet cell metaplasia and mucus hypersecretion, and proteolytic damage of airway walls [41, 42, 45, 53–56]. Further, it was shown that increased NE activity can aggravate the basic CF ion transport defect via proteolytic degradation of CFTR and cleavage activation of ENaC [6, 27, 36]. On the other hand, NE can contribute to bacterial killing and may thus have important protective functions in host defense [3, 22]. Therefore, complementary studies in preclinical models of CF lung disease are needed to define the relative roles of these disease-promoting versus protective functions of NE and potentially other proteases in the in vivo pathogenesis of CF lung disease.

## Review

### NE is implicated in airway inflammation, mucus hypersecretion, and structural lung damage in mice with CF-like lung disease

For a systematic analysis of the diverse functions of NE in the complex in vivo pathogenesis, a recent study made use of the βENaC-Tg mouse as an established model of CF lung disease. The βENaC-Tg mouse features airway surface dehydration and reduced mucus clearance characteristic of CF and phenocopies key features of CF lung disease including early-onset airway mucus plugging, spontaneous bacterial infection, chronic inflammation, and structural lung damage [29, 30, 34, 35, 57, 61]. To determine the in vivo role of NE in CF-like lung disease, βENaC-Tg mice were crossed with NE-deficient (NE^−/−^) mice [3] and the impact of genetic deletion on these pulmonary disease phenotypes was determined. This classical candidate gene approach provided several novel insights into the pathogenesis of early CF-like lung disease that may support the development of novel therapies [15].

**Fig. 1:**
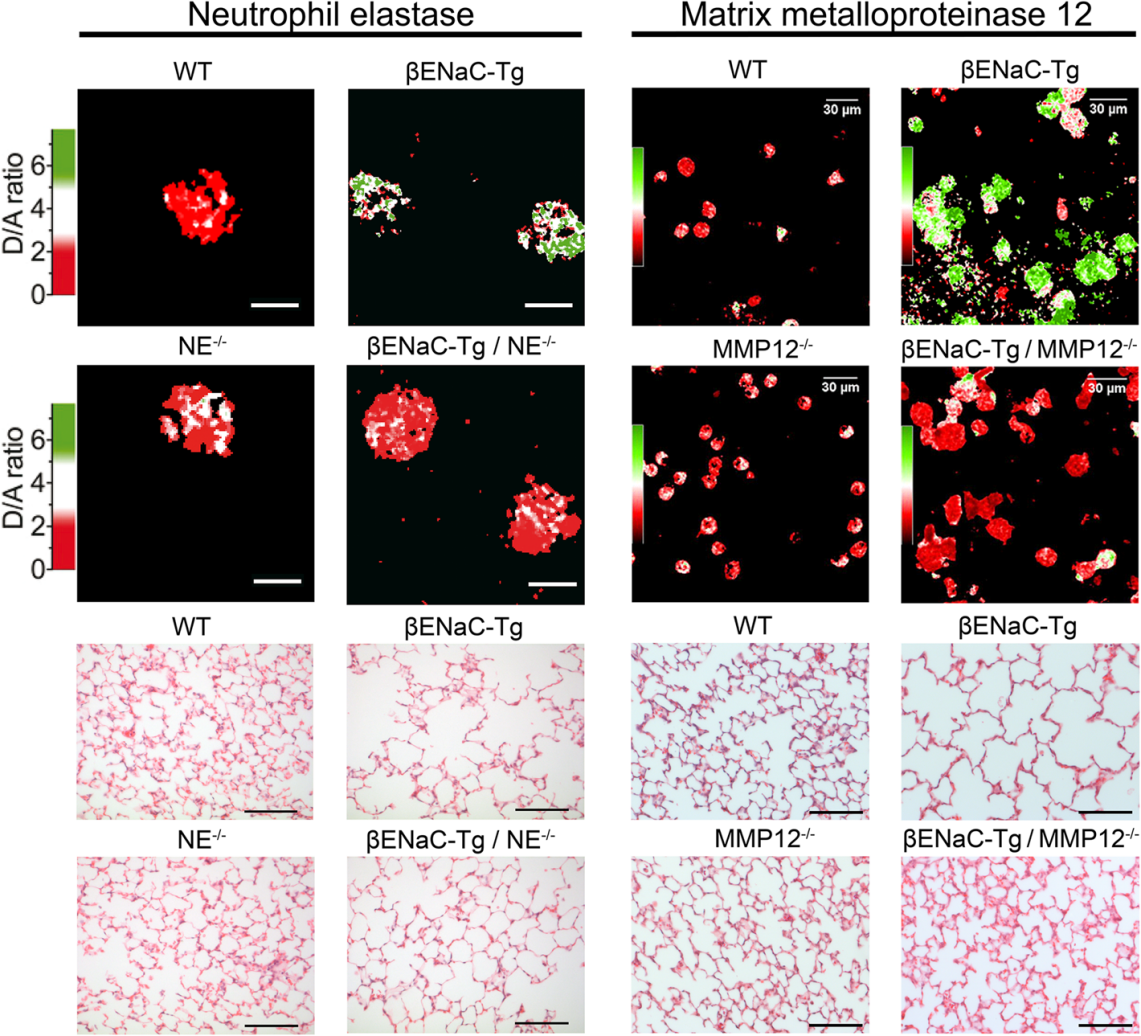
Neutrophil elastase (NE) and matrix metalloproteinase 12 (MMP12) activity is increased at the surface of bronchoalveolar neutrophils and macrophages and is associated with structural lung damage in βENaC-Tg mice. Protease activity was measured on the surface of neutrophils from bronchoalveolar lavage (BAL) using a lipidated FRET reporter for NE (NEmo-2) and representative ratio images calculated from donor and acceptor fluorescence are shown (*left upper panels*). NEmo-2 detects increased NE activity (*green color*) on neutrophils from βENaC-Tg compared to wild-type (WT) mice, and the specificity of the NEmo-2 FRET signal is confirmed by genetic deletion of NE (NE^−/−^ and βENaC-Tg/NE^−/−^ mice). Representative morphology of distal airspaces shows that increased NE activity on BAL neutrophils is associated with airspace enlargement and destruction in βENaC-Tg mice that is substantially reduced by genetic deletion of NE (*left lower panels*). Corresponding experiments using a lipidated FRET reporter for MMP12 (LaRee-1) show activity (*green color*) on macrophages from βENaC-Tg mice, but not from wild-type (WT) mice or mice that lack MMP12 (MMP12^−/−^ and βENaC-Tg/MMP12^−/−^ mice) (*right upper panels*). Representative morphology of lung sections from WT, βENaC-Tg, MMP12^−/−^, and βENaC-Tg/MMP12^−/−^ mice demonstrates that increased MMP12 activity on BAL macrophages also contributes to structural lung damage (*right lower panels*). Reprinted from [15, 52] with permission from the American Thoracic Society

First, the cross of these mouse models demonstrated that genetic deletion of NE results in a substantial reduction of neutrophils in BAL from βENaC-Tg mice confirming an important role of this protease in neutrophilic airway inflammation [15]. In this context, as described in more detail below, we found high levels of active NE on the surface of BAL neutrophils from βENaC-Tg mice (Fig. 1). We therefore speculate that membrane-associated NE activity plays an important role in the transmigration of neutrophils from the blood to the airway lumen and that this mechanism is a critical component of the pro-inflammatory function of NE in CF-like lung disease [15].

Next, these studies showed that NE is a potent trigger of goblet cell metaplasia and mucus hypersecretion in airways from βENaC-Tg mice. In fact, goblet cell metaplasia and increased expression of secreted mucins (*Muc5ac* and *Muc5b*) characteristic of βENaC-Tg mice [35] were completely abrogated in NE-deficient βENaC-Tg mice (βENaC-Tg/NE^−/−^) [15]. Surprisingly, even in the absence of mucus hypersecretion, βENaC-Tg/NE^−/−^ mice were not protected from the development of severe airway mucus obstruction. However, measurements of the airway mucus concentration demonstrated that the mucus was dehydrated to similar levels in βENaC-Tg/NE^−/−^ mice compared to βENaC-Tg littermates. These results suggest that airway surface dehydration may be sufficient to slow clearance of constitutively secreted mucus and trigger mucus plugging even in the absence of mucus hypersecretion [15].

In addition, these studies provided important information on the in vivo role of NE in antibacterial host defense. Mucociliary dysfunction in neonatal βENaC-Tg mice is associated with spontaneous bacterial infection dominated by species from the oropharyngeal flora [29]. In previous studies, lack of NE was shown to aggravate acute infection with *P. aeruginosa* in mice [3, 22]. On the other hand, high levels of NE activity were shown to cleave chemokine receptors on leukocytes compromising their ability to kill bacteria [20]. In this context, it was important to test the impact of genetic deletion of NE on the spontaneous airway infection of βENaC-Tg mice. These studies showed that lack of NE does not exacerbate bacterial infection in βENaC-Tg mice indicating that other innate and/or adaptive defense mechanisms [2] are sufficient to contain bacterial growth in the airways [15].

Finally, these studies demonstrated that NE is implicated in emphysema-like structural lung damage characteristic of βENaC-Tg mice [35, 57]. In contrast to patients with CF [50, 58], mice with chronic neutrophilic airway disease develop emphysema rather than bronchiectasis [35, 47, 57]. This species difference is probably related to anatomical differences including a substantially lower number of airway branching in the mouse compared to the human lung that may result in a faster spillover of damaging factors from the conducting airways to the distal airspaces in mice. Nevertheless, it was found that genetic deletion of NE leads to a significant reduction (~50 %) of distal airspace enlargement and alveolar destruction in βENaC-Tg mice (Fig. 1) [15]. Despite the species differences mentioned above, these results support the concept that NE plays a critical role in the in vivo pathogenesis of structural lung damage associated with neutrophilic airway inflammation.

To elucidate the localization of tissue damaging protease activity, highly sensitive FRET reporters were employed that can discriminate between free (NEmo-1) and membrane-bound (NEmo-2) NE activity in CF-like lung disease (Fig. 1) [16, 23]. Similar to infants and young children with CF, βENaC-Tg mice exhibit a moderate airway neutrophilia with 5–30 % of neutrophils in BAL fluid [30, 49, 61]. Using the NEmo FRET reporters, we found that NE activity is invariably increased on the surface of BAL neutrophils from βENaC-Tg mice compared to wild-type controls (Fig. 1), whereas no free NE activity was detected in cell-free BAL supernatant [15]. In addition, we found that the activity of purified NE is potently inhibited by BAL supernatant from βENaC-Tg mice indicating that NE secreted from activated neutrophils into the extracellular compartment is inhibited by a robust antiprotease shield [15]. In CF patients with advanced lung disease and higher neutrophil counts (>80 %), NEmo-2 also detected higher levels of NE activity on the surface of sputum neutrophils. Further, free NE activity was increased in sputum supernatant from patients with CF compared to healthy controls as expected from previous studies [15, 37, 46]. When viewed in combination, these results suggest (i) that free NE activity is inhibited as long as the antiprotease shield composed of NE inhibitors such as α1-antitrypsin and secretory leukocyte protease inhibitor (SLPI) is not overwhelmed [26, 42, 43] and (ii) that surface-bound NE activity may play a critical role in tissue damage, even in early CF lung disease with moderate airway neutrophilia [15].

### MMP12 contributes to structural lung damage in mice with CF-like lung disease

Interestingly, quantitative phenotyping of the cross of βENaC-Tg mice with NE^−/−^ mice also revealed substantial residual alveolar destruction in double-mutant βENaC-Tg/NE^−/−^ mice indicating that increased NE activity only accounts for ~50 % of structural lung damage and that other factors contribute to emphysema formation in βENaC-Tg mice [15]. Spurred by this observation, whole-genome expression profiling of the lung tissues was used as a bottom-up approach to search for candidate genes and pathways responsible for residual emphysema formation in βENaC-Tg mice. This unbiased approach identified *Mmp12*, but no other candidates previously implicated in emphysema formation, as a strongly upregulated gene in the lungs from βENaC-Tg mice [52]. Matrix metalloproteinase 12 (MMP12) is a matrix metalloproteinase with elastolytic capacity [48] that is secreted by activated macrophages and has been implicated in emphysema pathogenesis in mouse models of COPD and asthma via several independent mechanisms including (i) degradation of the extracellular matrix, (ii) proteolytic inactivation of antiproteases such as α1-antitrypsin, and (iii) proteolytic activation of proinflammatory cytokines including TNFα [10, 21, 60]. Interestingly, MMP12 is also a signature gene of alternatively activated macrophages (AAM) and the majority of genes that were differentially upregulated in the lungs from βENaC-Tg mice (*Alox15*, *Arg1*, *Chi3l3*, *Chi3l4*, *Mgl2*, *Retnla*) belong to this AAM signature [18, 28]. These results suggest that the microenvironment of mucostatic CF-like airways triggers alternative macrophage activation, which in turn results in upregulation of MMP12 [52].

To validate the pathogenetic relevance of MMP12 in emphysema formation in βENaC-Tg mice, the temporal relationship between elevated MMP12 expression and structural lung damage was investigated by several independent approaches including (i) MMP12-specific FRET reporters to localize its activity in the lung [11], (ii) genetic deletion of MMP12, and (iii) pharmacological inhibition of MMP activity in βENaC-Tg mice [52]. These studies demonstrated a strong temporal association of increased *Mmp12* expression with distal airspace enlargement and destruction. First, it was shown that these emphysema-like features are substantially (~50 %) reduced by deletion of MMP12 in βENaC-Tg mice (Fig. 1). Similar effects were observed when βENaC-Tg mice were treated with the MMP inhibitor GM 6001. However, in contrast with the findings in βENaC-Tg/NE^−/−^ mice, neither genetic deletion nor pharmacological inhibition of MMP12 reduced airway inflammation or goblet cell metaplasia in βENaC-Tg mice indicating that MMP12 is not essential in the in vivo pathogenesis of CF-like airway diseases [52]. Similar to findings for NE on activated neutrophils, studies using FRET reporters capable of detecting cell membrane-bound (LaRee-1) and free (LaRee-5) MMP12 activity [11, 23] showed that the activity of this protease is significantly increased on the surface of BAL macrophages (Fig. 1), but not in BAL fluid from βENaC-Tg mice [52]. These results indicate that the zymogen form of MMP12 is activated at the macrophage surface and that secreted MMP12 is inhibited by antiproteases in the extracellular milieu [42]. Collectively, these studies identified MMP12 secreted by activated macrophages as an additional protease contributing to the in vivo pathogenesis of structural lung damage in CF-like lung disease. Further, these studies corroborate the concept that protease activity on the surface of activated inflammatory cells, via direct contact to the extracellular matrix, plays an important role in structural lung damage associated with chronic airway inflammation [52].

Following biological validation of a candidate gene in a mouse model, it remains critical to determine its respective role in human disease. Previous studies have implicated MMP12 in the pathogenesis of COPD and asthma. Specifically, it was shown that elevated levels of MMP12 in the sputum are associated with emphysema severity in COPD [8, 12, 38] and that a functional variant in the *MMP12* promoter (rs2276109) [25, 59] is associated with a beneficial effect on lung function in children with asthma, as well as a reduced risk for adult smokers to develop COPD [24]. In comparison, current knowledge on the role of MMP12 in CF lung disease remains limited [14]. One study detected alternatively activated macrophages in BAL and demonstrated an inverse relationship with lung function in patients with CF; however, MMP12 levels were not determined [39]. More recently, the development of the LaRee FRET reporters enabled measurements of MMP12 activity in BAL samples from patients with CF [52]. Similar to the results obtained in βENaC-Tg mice (Fig. 1), these studies detected increased activity of MMP12 at the macrophage surface even in children with CF with early lung disease. The functional relevance of this finding is supported by a genetic association study that investigated the impact of SNP in *MMP12* on lung function in a cohort of 442 patients with CF [52]. This study showed that the SNP in the MMP12 promoter (rs2276109), as well as a tightly linked SNP (rs737693), is positively associated with longitudinal lung function (FEV_1_ % predicted) in patients with CF. Taken together, these translational studies provide initial evidence that proteolytic activity of MMP12 secreted by macrophages that are activated on mucostatic airway surfaces may contribute to the pathogenesis of structural lung damage and lung function decline in patients with CF [52].

## Conclusions

In summary, the cross of βENaC-Tg mice with NE^−/−^ mice demonstrates that NE is implicated in the in vivo pathogenesis of several key features of CF-like lung disease including the modulation of neutrophilic airway inflammation, induction of goblet cell metaplasia and mucin hypersecretion, and structural lung damage [15]. Further, whole-genome expression profiling as an unbiased bottom-up approach led to the identification of MMP12 released from activated macrophages as an important contributor to tissue damage in CF-like lung disease [52]. Importantly, genetic deletion of NE and MMP12 did not exacerbate spontaneous airway infection in βENaC-Tg mice. These data support that these proteases are promising targets for novel anti-inflammatory and tissue-protective therapies in CF. Interestingly, localization of protease activity with sensitive FRET reporters showed that the activities of both NE and MMP12 are invariably increased on the surface of activated neutrophils and macrophages, respectively, even under conditions when “free” activity of these secreted proteases is absorbed by an intact antiprotease shield [15, 52]. These results suggest that membrane-bound protease activity may play a critical role in airway damage and that NE and MMP12 may have to be inhibited at the surface of inflammatory cells to achieve maximal therapeutic effects. In this context, translational studies in clinical specimens (BAL and sputum) from patients with CF indicate that lipidated FRET reporters designed to measure protease activity at the surface of inflammatory cells, such as NEmo-2 and LaRee-1 [11, 16], may be sensitive novel tools to identify CF patients with the greatest risk to develop severe lung damage. However, additional studies in a larger number of patients that address the relationship with clinical indices of lung disease severity, such as lung function and imaging endpoints, will be required to determine the value of membrane-bound protease activity as an inflammation biomarker in CF and potentially other neutrophilic airway diseases. Of note, studies in βENaC-Tg mice also demonstrated that genetic or pharmacological inhibition of NE and MMP12 does not prevent dehydration-induced airway mucus plugging in vivo indicating that additional rehydration therapies such as osmolytes [19] or modulators of epithelial ion channels including CFTR, ENaC, or alternative Cl^−^ channels [33] may be required for effective treatment of airway mucus plugging in CF.
